# Supplementary material for: Splice-Junction-Based Mapping of Alternative Isoforms in the Human Proteome
Source: Cell Rep. Author manuscript; Available in PMC 2020 Jan 15. (PMC6961840; doi:10.1016/j.celrep.2019.11.026)
Supplement: 2 [file NIHMS1546469-supplement-2.zip › DF1/PXD006675/PulmonaryVein/PulmonaryVein_4_TTN_RVEAEPAEEVTIMEEK.pdf]

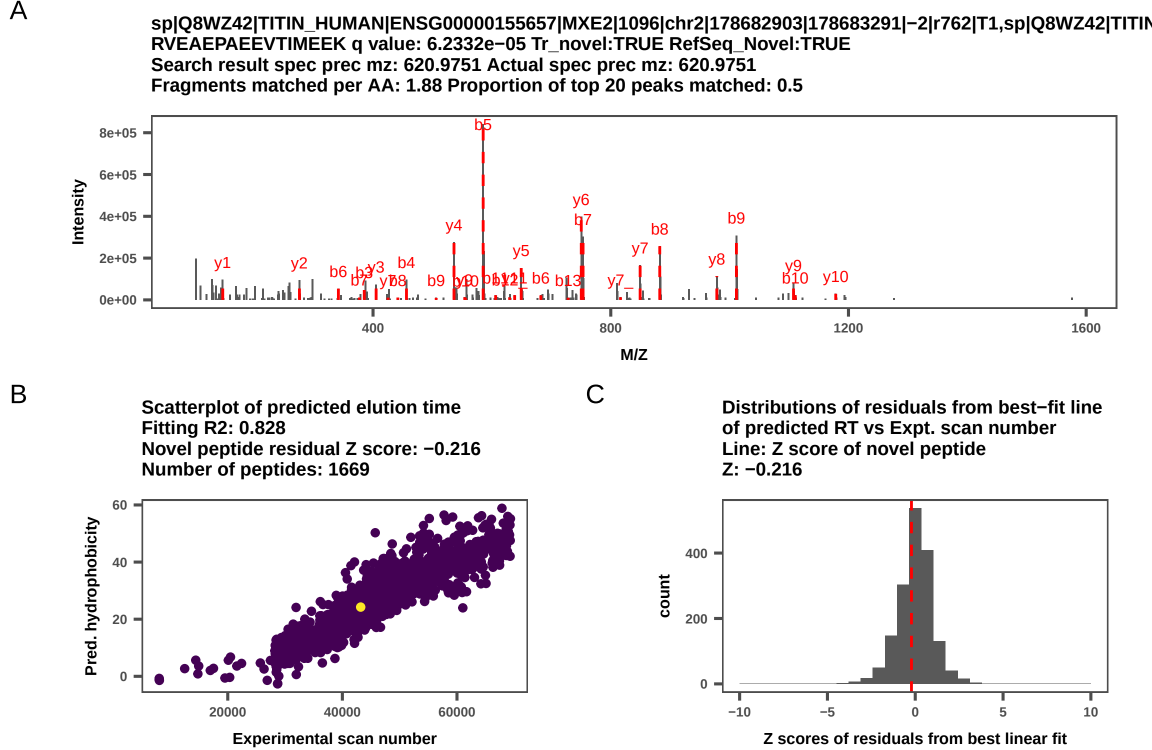
A
sp|Q8WZ42|TITIN_HUMAN|ENSG00000155657|MXE2|1096|chr2|178682903|178683291|−2|r762|T1,sp|Q8WZ42|TITIN
RVEAEPAEEVTIMEEK q value: 6.2332e−05 Tr_novel:TRUE RefSeq_Novel:TRUE
Search result spec prec mz: 620.9751 Actual spec prec mz: 620.9751
Fragments matched per AA: 1.88 Proportion of top 20 peaks matched: 0.5
Intensity
8e+05
6e+05
4e+05
2e+05
0e+00
b5
y6
b7
b9
y4
b8
y5
y7
y8
y1
y2
b4
b6
y9
b10
y10
b9
b13
M/Z
400
800
1200
1600
B
Scatterplot of predicted elution time
Fitting R2: 0.828
Novel peptide residual Z score: −0.216
Number of peptides: 1669
Pred. hydrophobicity
60
40
20
0
20000
40000
60000
Experimental scan number
C
Distributions of residuals from best−fit line
of predicted RT vs Expt. scan number
Line: Z score of novel peptide
Z: −0.216
count
400
200
0
−10
−5
0
5
10
Z scores of residuals from best linear fit
